# Fermentation Characteristics of Fermented Milk with *Streptococcus thermophilus* CICC 6063 and *Lactobacillus helveticus* CICC 6064 and Volatile Compound Dynamic Profiles during Fermentation and Storage

**DOI:** 10.3390/molecules29061257

**Published:** 2024-03-12

**Authors:** Xiaoxin Zhao, Yuanyuan Ge, Xuejian Yu, Chong Liu, Haizhi Li, Xi Wang, Su Yao

**Affiliations:** 1 China Center of Industrial Culture Collection, China National Research Institute of Food and Fermentation Industries Co., Ltd., Beijing 100015, China; 13654680363@163.com (X.Z.); lucy@china-cicc.org (Y.G.); yuxuejian@china-cicc.org (X.Y.); liuchong@china-cicc.cn (C.L.); li_haizhi@163.com (H.L.); wangxi1822@163.com (X.W.); 2 College of Biological Sciences and Biotechnology, Beijing Forestry University, Beijing 100083, China

**Keywords:** *Streptococcus thermophilus*, *Lactobacillus helveticus*, fermented milk, fermentation characteristics, solid-phase microextraction and gas chromatography–mass spectrometry (SPME-GC-MS)

## Abstract

The lactic acid bacteria *Streptococcus thermophilus* and *Lactobacillus helveticus* are commonly used as starter cultures in dairy product production. This study aimed to investigate the characteristics of fermented milk using different ratios of these strains and analyze the changes in volatile compounds during fermentation and storage. A 10:1 ratio of *Streptococcus thermophilus* CICC 6063 to *Lactobacillus helveticus* CICC 6064 showed optimal fermentation time (4.2 h), viable cell count (9.64 log10 colony-forming units/mL), and sensory evaluation score (79.1 points). In total, 56 volatile compounds were identified and quantified by solid-phase microextraction and gas chromatography–mass spectrometry (SPME-GC-MS), including aldehydes, ketones, acids, alcohols, esters, and others. Among these, according to VIP analysis, 2,3-butanedione, acetoin, 2,3-pentanedione, hexanoic acid, acetic acid, acetaldehyde, and butanoic acid were identified as discriminatory volatile metabolites for distinguishing between different time points. Throughout the fermentation and storage process, the levels of 2,3-pentanedione and acetoin exhibited synergistic dynamics. These findings enhance our understanding of the chemical and molecular characteristics of milk fermented with *Streptococcus thermophilus* and *Lactobacillus helveticus*, providing a basis for improving the flavor and odor of dairy products during fermentation and storage.

## 1. Introduction

Fermented milk is produced by the addition of suitable bacteria to usually heat-treated animal milk, followed by incubation to reduce the pH, with or without coagulation pretreatment [1]. Lactic acid bacteria (LAB), recognized as safe for consumption (GRAS), are extensively employed in the industrial production of fermented dairy products, wine, bread, sour beer, and vegetables [2]. Fermented foods can modulate gut microbiota and influence the host immune system through the release of nutrients and metabolites during the fermentation process [3]. *Streptococcus thermophilus* is related to other LABs, such as *Lactococcus lactis*, which is the most important industrial starter culture widely used in the dairy industry [4]. It has the ability to metabolize lactose into exopolysaccharides, vitamins, and several flavor compounds [5]. *Lactobacillus helveticus* is a widely used starter culture in the production of yogurt, Italian cheeses, and Swiss cheeses, exhibiting probiotic properties such as gastrointestinal survival, epithelial cell adhesion, and pathogen antagonism [6,7]. It also acts as an effective inhibitor of angiotensin-converting enzyme, providing potential benefits for managing hypertension [8]. Moreover, fermented milk with *L. helveticus* has been found to positively impact calcium metabolism [9]. *S. thermophilus* and *L. helveticus* can cooperate to enhance each other’s growth, accelerate acidification, increase exopolysaccharides (EPS) synthesis, and improve the texture, rheological properties, and flavor of fermented milk during storage [10,11,12].

During fermentation, milk components undergo conversion into various metabolites, including volatile and non-volatile flavor compounds [13]. These aromatic compounds can be categorized into different groups based on their chemical structures, such as carbonyl compounds, alcohols, acids, esters, hydrocarbons, aromatic compounds, and compounds containing sulfur [14]. The primary volatile chemicals responsible for the desired scent in yogurt are acetaldehyde, diacetyl, acetone, acetic acid, and 2-butanone [15]. Although present in small amounts, these compounds play a significant sensory role in fermented milk. It is worth noting that flavor substances undergo changes not only during fermentation but also during storage [16]. And *L. helveticus* is important in the dairy industry for reducing bitterness and imparting a characteristic flavor to cheese [12].

The gas chromatography–mass spectrometry (GC-MS) method is widely used to examine the volatile chemicals. Furthermore, solid-phase microextraction (SPME), which enables the quick isolation of volatile chemicals from both solid and liquid matrices, can be used to examine a small number of samples [17]. Metabolic profiles of fermented milk from *S. thermophilus* and *L. delbrueckii* ssp. *bulgaricus* have been reported [18]. The combined effects of *S. thermophilus* and *L. helveticus* on product quality, including flavor and fermentation characteristics, during fermentation and storage have not, however, been systematically studied. In a previous study, *S. thermophilus* CICC 6063 and *L. helveticus* CICC 6064 have been used in the production of naked oat fermented beverage [19]. Thus, this study aims to investigate how different inoculum ratios of *S. thermophilus* CICC 6063 and *L. helveticus* CICC 6064 affect fermentation characteristics and explore the flavor profile during the entire process, particularly under the optimal ratio. This is important for understanding the relationship between *S. thermophilus* and *L. helveticus* and their relative contributions to the fermentation process, with the aim of providing a reference for the development and use of starter cultures.

## 2. Results

### 2.1. Physicochemical Characteristics of Fermented Milk in Different Inoculum Ratios

**Figure 1:**
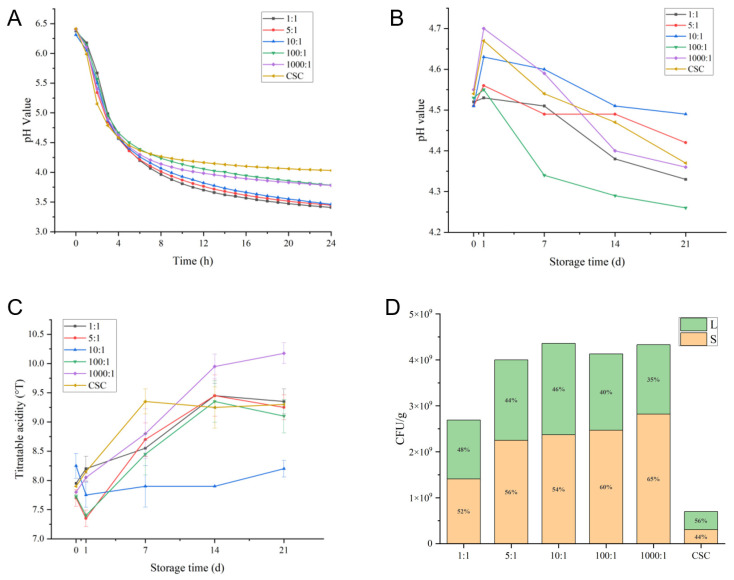
Characteristics of fermented milk with varying strain proportions: (**A**) 24 h pH monitoring results during milk fermentation by varying ratios of *S. thermophilus* CICC 6063 and *L. helveticus* CICC 6064 co-cultures, including 1:1, 5:1, 10:1, 100:1, and 1000:1 (*S. thermophilus* to *L. helveticus*) and commercial starter culture (CSC). (**B**) Variations in fermented milk pH during storage. (**C**) Variations in fermented milk titration acidity during storage. (**D**) Counting viable bacteria in fermented milk stored for 1 day (S: *Streptococcus*, L: *Lactobacillus*).

Various parameters, including fermentation time, pH, titratable acidity (TA), and viable bacteria count, were measured to assess the fermentation characteristics of *S. thermophilus* CICC 6063 and *L. helveticus* CICC 6064 in the fermented milk samples. The pH level of the fermented milk was continuously monitored for 24 h in Figure 1A. All samples in the six groups reached the casein isoelectric point within 4 to 5 h, with the sample containing a 5:1 and 10:1 ratio fermenting the fastest, at 4.2 h. The 1000:1 fermented milk took the longest, at 4.6 h, to complete fermentation. The titrated acidity at the end of fermentation ranged from 77 to 83 °T. The pH and TA of the samples in each group changed over time (Figure 1B,C). After post-ripening, the pH values increased compared to the end point of fermentation, but gradually decreased during the 21-day storage period, similar to the TA results. The 10:1 sample had the smallest changes in both pH and TA, with values of 0.14 and 4.5, respectively. In the later stages of storage, the pH and TA values of the samples remained relatively stable, ranging from 4.2 to 4.6 and 82 to 104 °T, respectively. The Figure 1D displays the viable bacteria count results of fermented milk produced using different inoculation ratios. Among the tested samples, the fermented milk made with a 10:1 ratio of CICC 6063 to CICC 6064 exhibited the highest total viable bacteria count (4.36 × 10^9^ CFU/mL). Moreover, the total viable bacteria count in fermented milk samples prepared using different proportions of the complex starter was found to be higher than that of the commercial starter culture.

### 2.2. Rheological Properties of Fermented Milk at Different Inoculum Ratios

**Figure 2:**
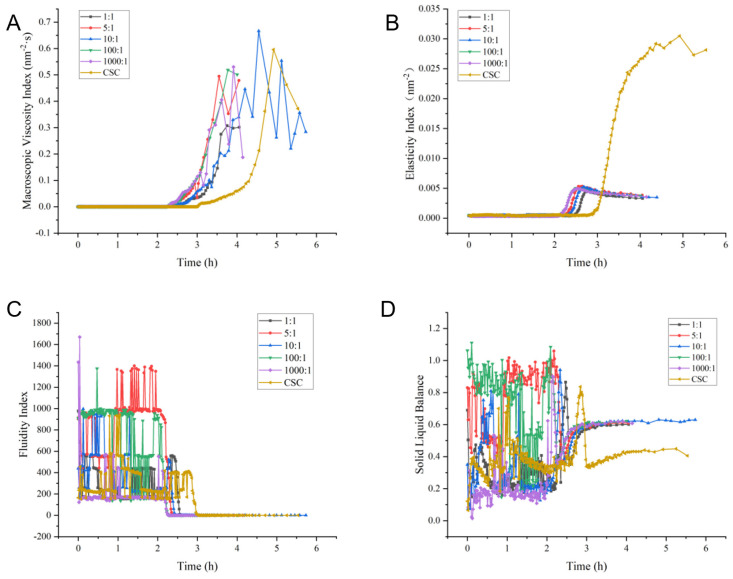
Rheological results of fermented milk prepared with different proportions of co-culture and commercial starter culture: (**A**) aacroscopic viscosity index (MVI) values; (**B**) elastic index (EI) values; (**C**) fluidity index (FI) values; (**D**) solid-liquid balance (SLB) values.

The MVI value in Figure 2A indicates the sample’s microscopic viscosity. The casein micelles reorganized into a gel structure over two hours as the pH decreased. The 10:1 ratio sample reached its highest viscosity at 4.5 h, near the end of fermentation. Figure 2B demonstrates the agreement between EI and MVI values. The fermented milk groups’ EI values stabilized after 2.5 h, indicating a stable gel structure formed. At a stable state, the control group exhibited a strong gel structure. The FI values of both groups experienced significant fluctuations before reaching the gel point, as illustrated in Figure 2C, indicating that the sample remained in a liquid state. The 1000:1 fermented milk group reached the inflection point first after two hours, resulting in a sharp decrease in the FI value. The solid and elastic properties of the fermented milk fell within the SLB range of 0 to 0.5. However, the fermented milk displayed liquid and viscous characteristics within the SLB range of 0.5 to 1. After three hours, the stable sample tended to become more liquid, while the control group tended to become more solid, as depicted in Figure 2D.

### 2.3. Sensory Characteristics of Fermented Milk at Different Inoculum Ratios

**Figure 3:**
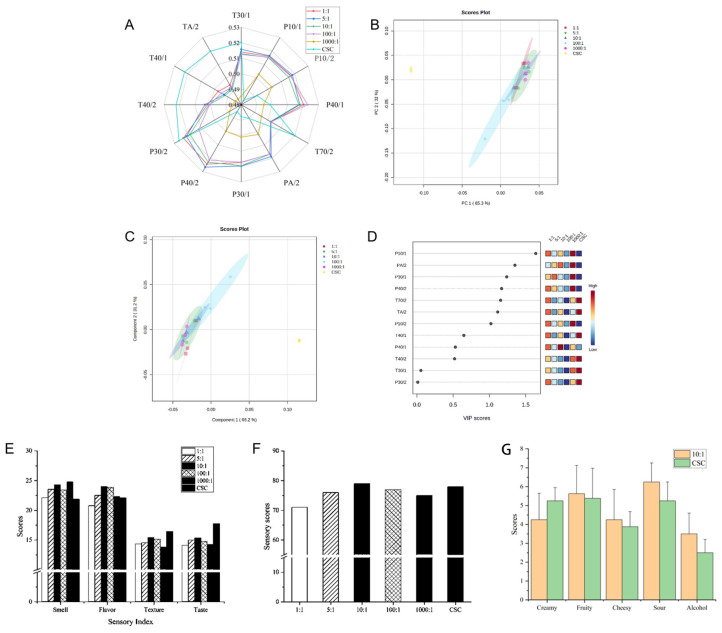
Electronic nose and sensory evaluation results of fermented milk prepared with different proportions of co-culture and commercial starter culture: (**A**) radar fingerprint chart of electronic nose; (**B**) principal component analysis (PCA); (**C**,**D**) scores by PLS-DA of E-nose data; (**E**) sensory scores of different sensory indexes; (**F**) total sensory scores of samples; (**G**) the average scores of sensory descriptors of 10:1 and CSC group.

The use of an electronic nose as a rapid assessment tool for volatile flavor compounds in fermented dairy products offers numerous benefits. Figure 3 presents the electronic nose data for fermented milk prepared using different cultures, highlighting the outcomes of the preparation. The radar map demonstrates that, except for the control group and the 1000:1 ratio, the flavor profiles of the remaining four groups were similar. Furthermore, certain sensors (P10/1, P30/2, P30/1, T30/1, P40/2, and PA/2) exhibited an enhanced response within these four groups, indicating the presence of significant amounts of organic and alcoholic compounds in the samples (Figure 3A). The PCA chart in Figure 3B clearly depicts the differences in flavor attributes among the six groups. The PLS-DA results indicate that PC1 and PC2 account for 65.2% and 31.2% of the sample variation, respectively, collectively explaining 96.4% of the total variance (Figure 3C). In Figure 3B,C, we used R2Y (goodness-of-fit) and Q2 (predictive ability parameter) to assess the accuracy and predictability of the PLS-DA model. The R2Y and Q2 values were 0.90 and 0.68, respectively, indicating good accuracy and predictability of the model. The PLS-DA VIP analysis identified several sensors, including P10/1, P30/1, P40/2, PA/2, T70/2, TA/2, and P10/2, that were found to have a significant representation of over 1 (Figure 3D).

A total of six sets of fermented milk samples were evaluated for their flavor, texture, aroma, and taste. In comparison to commercial starter cultures, the combined fermented milk exhibited improved flavor and aroma, as depicted in Figure 3E. The fermented milk with a 10:1 ratio achieved the highest overall score, closely resembling the sensory characteristics of the imported commodity starter, as shown in Figure 3F. Our findings indicate that the ratio of *S. thermophilus* CICC 6063 to *L. helveticus* CICC 6064 has a significant impact on the production of volatile flavor compounds and the sensory quality of fermented milk. The 10:1 group had relatively higher scores in smell and flavor, but lower texture and taste characteristics compared with the CSC group. We recommend using a ratio of 10:1 for producing fermented milk with desirable flavor and storage stability. The sensory characteristics of the 10:1 and CSC groups were evaluated using quantitative descriptive analysis (QDA). The primary flavor attributes were defined by the first two sensory characteristics, with the 10:1 group exhibiting predominantly sour and fruity flavors, while the CSC group showed fruity and creamy flavors. It has been reported that increasing sourness intensity can mask the creamy flavor [20]. Additionally, the 10:1 group demonstrated stronger fruit, cheese, sour, and alcoholic flavors. In dairy products, the flavors of alcohol and fruit primarily originate from ester compounds [21]. The higher levels of ester compounds, particularly ethyl esters with alcoholic and fruity flavors, in the 10:1 group align with the results of volatile flavor compound determinations.

### 2.4. Volatile Compounds Profiles during Fermentation and Storage

The odor and flavor formation in milk products involves the breakdown of chemical components through protein, glycolysis, and lipolysis. In this study, a 10:1 co-culture sample was used to analyze volatile compounds at different time points (0 h (F), 2 h (F), 4 h (F), 0 d (S), 1 d (S), 7 d (S), 14 d (S), and 21 d (S)) during fermentation and storage. Table 1 presents the retention index (RI) of the DB-Wax column, which identified a total of 56 compounds. These compounds include ten acids, ten aldehydes, ten ketones, eleven alcohols, five esters, and ten aromatic carbohydrates. Mass spectrometry was used to identify these compounds, and their relative intensities (RIs) were calculated using an alkane mixed standard and cross-referenced with published research.

During the fermentation and storage of fermented milk, ten different acids were detected. Notably, the short-chain fatty acids acetic, butanoic, and hexanoic acid were found to increase significantly, particularly towards the end of fermentation, reaching maximum concentrations of 39.44, 50.45, and 90.84 µg/L, respectively. Additionally, octanoic acid exhibited a fluctuating pattern, initially rising, then declining, and finally rising again. The highest concentration of octanoic acid, 14.34 µg/L, was observed at 0 d (S). In summary, the fermented milk contained a diverse range of acids in varying concentrations, which underwent changes throughout the fermentation and storage phases.

During fermentation and storage, ten aldehydes were identified. The highest concentration was observed at 4 h (F) and 0 d (S). Acetaldehyde, in particular, exhibited high levels during fermentation, reaching its peak at 14 d (S) before gradually decreasing to 74.39 µg/L at the completion of the process. Additionally, benzaldehyde was detected at a concentration of 1.94 µg/L at the end of fermentation. Lastly, 2-methyl-2-butenal (0.31–1.11 µg/L) was detected during the later stages of storage.

Ketone levels were higher in fermented milk compared to other materials during production and storage. Ten distinct types of ketones were identified. The concentration of 2-heptanone was highest during the early stages of fermentation (108.5 µg/L). The acetoin concentration increased and peaked at 0 d (S) during fermentation, then decreased at 1 d (S) and increased again. After fermentation, the mass concentration of 2,3-butanedione was 111.51 µg/L, with some variations observed during storage. After 21 days of storage, the concentration of 2,3-pentanedione increased to 51.36 µg/L. The concentration of 2-nononone increased again after decreasing during fermentation, following 21 days of storage. After seven days of storage, 4-methyl-2-hexanone was detected, with a mass concentration ranging from 1.02 to 3.03 µg/L. The total content of ketones, including 2,3-butanedione, acetoin, acetone, 2-heptanone, and 2-nononone, among others, was high (32.44–135.28 µg/L, 58.65–231.14 µg/L, 2.51–42.99 µg/L, 28.79–41.34 µg/L, and 1.46–7.79 µg/L, respectively).

Eleven alcohols, including 3-pentanol, 2-hexanol, and methyl alcohol, were detected in the fermented milk. Initially, methanol had a high concentration of 97.33 µg/L during fermentation, which later decreased. The maximum concentration of 3-pentanol was 6.29 µg/L at the end of fermentation. The concentration of 1,2-ethanediol steadily increased during storage, ranging from 0.43 to 0.83 µg/L. On the other hand, the content of 1-propene-1-thiol ranged from 0.05 to 0.88 µg/L and gradually decreased after fermentation.

At the start of the process, ethyl hexanoate was found at a concentration of 2.12 µg/L among the esters and aromatic carbohydrates. The concentration of *p*-xylene varied during different stages, reaching its peak at 2 h (F). Throughout storage, the content of *p*-xylene ranged from 8.74 to 29.83 µg/L. Styrene was detected at concentrations ranging from 1.80 to 22.01 µg/L throughout the entire procedure.

### 2.5. Analysis of Volatile Compounds during Fermentation and Storage

**Figure 4:**
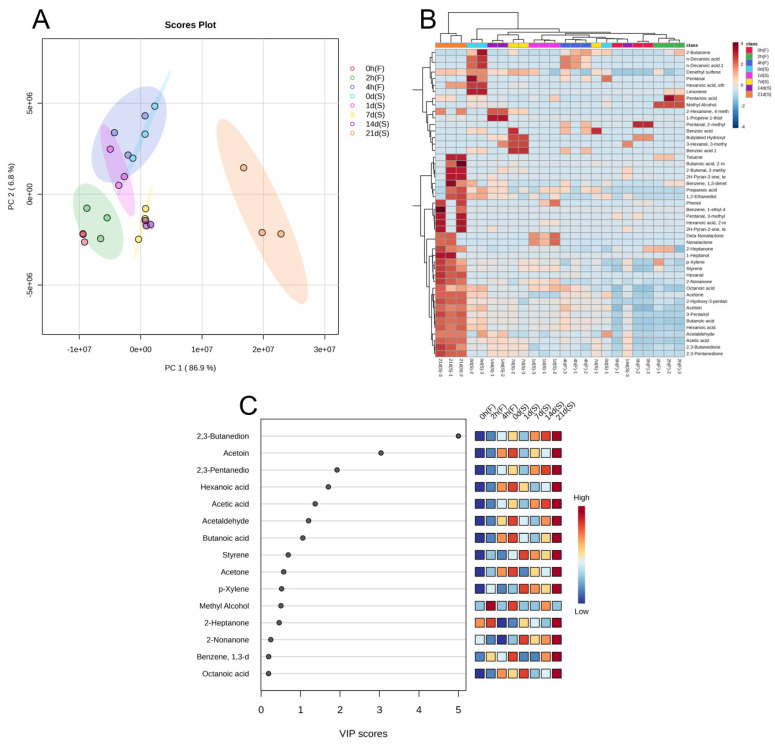
Differences in volatile metabolome of 10:1 *S. thermophilus* to *L. helveticus*-fermented milk at different time points during fermentation and storage. (**A**) The principal component analysis (PCA) score plot revealed the volatile metabolomes. These time points included the initial point before fermentation (0 h (F)), two intermediate time points (2 h (F), 4 h (F)), the termination point of fermentation (0 d (S)), and different stages of refrigerated storage (1 d (S), 7 d (S), 14 d (S), and 21 d (S)). (**B**) Heatmap of identified volatile metabolites in each group. (**C**) VIP result diagram of PLS-DA.

Principal component analysis (PCA) is a statistical technique used to reduce the dimensionality of variables while preserving their original information [22]. In the principal component loading diagram, a closer proximity between time points and aroma components indicates a higher correlation. Figure 4A displays the principal component score chart for fermented milk samples during fermentation and storage, which is divided into four distinct regions. The time points 0 h (F) and 2 h (F) overlap, as do the 4 h (F), 0 d (S), and 1 d (S) time points, suggesting similar flavor profiles. However, 21 d (S) stands alone with distinct characteristics, while 1 d (S) and 7 d (S) exhibit similar flavors. Principal component analysis (PCA) confirms that samples at the late storage stage differ significantly from other time points. Figure 4B shows how samples collected at different times of day form unique clusters, while Figure 4C demonstrates significant variation in the contents of seven volatile flavor substances. These substances include butanoic acid, hexanoic acid, acetic acid, acetaldehyde, 2,3-butanedione, acetoin, and 2,3-pentanedione (VIP > 1).

## 3. Discussion

The choice of suitable starter cultures is crucial for producing high-quality and consistent fermented milk products. Previous research has shown that *S. thermophilus* CICC 6063 exhibits a high fermentation rate. In this study, we aimed to investigate the fermentation characteristics of fermented milk produced using different ratios of *S. thermophilus* CICC 6063 to *L. helveticus* CICC 6064. Furthermore, we assessed the production and storage stability of volatile flavor compounds under the optimal ratio.

In selecting a starter with optimal fermentation properties, factors such as fermentation time, pH, titratable acidity (TA), and live cell count are considered important [23]. In this study, a representative sample consisting of a 10:1 ratio was observed to reach the isoelectric point of casein after 4.2 h, indicating the potential to decrease fermentation time through the strategic combination of strains. The pH values of all samples rapidly declined within the initial 2 to 4 h post-inoculation, which is consistent with previous findings [24]. During the 21 days of storage at 4 °C, the pH and titratable acidity of the yogurt samples were significantly influenced by the storage time. The pH decreased while the titratable acidity increased, as observed in a study by Shah N. on fermented milk co-cultured with *L. helveticus* and *S. thermophilus* [25]. Additionally, samples with a 10:1 ratio and a commercial starter exhibited a more pronounced weak post-acidification effect, potentially extending the product’s shelf life.

Microrheology can reflect the rheological properties of fermented milk over time, which in turn affect the texture and taste of the product. The development of viscosity modulus can be measured directly using the MVI index, which captures changes in the lost modulus within a specific time period, providing insights into the variations in viscosity [26]. At the 2 h mark, co-culture samples showed an increase in viscosity, potentially due to the separation of casein micelles, development of emulsion gels, and swift viscosity alteration [27]. The MVI of each fermented milk sample peaked and transitioned into a phase of heightened viscosity [28]. An increase in the experimental investigation (EI) value indicates a stronger gel structure and improved stability in the sample. The gel strength in co-culture samples was observed to be lower compared to the control group, possibly due to the efficient proteolytic system of *L. helveticus* [29]. The fluidity index (FI) quantifies the velocity of particle movement within the system [30]. The SLB analysis revealed that acid production conditions influenced fermentation time variation, with the control group having the lowest SLB value at approximately 0.4. Co-culture samples showed a slightly higher SLB value of around 0.6, while the 10:1 group had a slightly higher SLB value compared to the other groups. Co-culture samples exhibited a higher liquid viscosity, while the control group had superior solid elasticity (CSC group).

**Figure 5:**
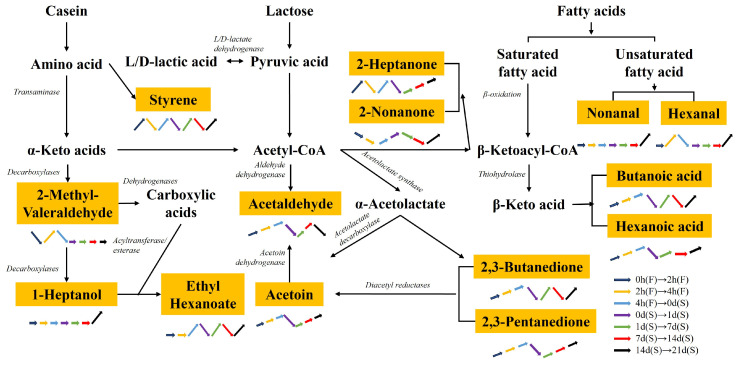
Metabolic pathway prediction and concentration changes in discriminatory volatile metabolites in this study.

The flavor of fermented milk is influenced by various volatile compounds, which undergo significant changes in types and concentrations during fermentation and storage, as depicted in Table 1. This study is the first to examine the flavor fingerprints of *S. thermophilus* and *L. helveticus* throughout fermentation and storage, revealing that the concentrations of the majority of flavor compounds peaked at the end of the fermentation process, indicating superior flavor characteristics compared to other fermentation stages. Furthermore, Figure 5 illustrates the metabolic pathways of discriminatory volatile metabolites, and their content fluctuations during fermentation and storage.

Carboxylic acids derived from lipolysis, proteolysis, or lactose fermentation in fermented milk enhance taste and contribute to sourness [17,31]. These acids, including methyl ketones, alcohols, esters, and aldehydes, have minimal impact on the overall flavor due to their high thresholds [32]. Among them, butanoic acid and hexanoic acid significantly alter the flavor of fermented milk and increase steadily during fermentation, reaching a peak at the end. After storage, there is a temporary decrease followed by a gradual rise. Hexanoic and heptanoic acids are likely released through lipolytic activity [33]. Hexanoic acid imparts a rancid, sweet cheese-like taste, and n-decanoic acid and acetic acid are also detected. Octanoic acid, ranging from 1.57 to 14.34 µg/L, can contribute a soapy taste [34]. Fermented milk containing hexanoic acid, octanoic acid, decanoic acid, and other fatty acids exhibits potential probiotic properties [35]. These fatty acids are present throughout fermentation and storage, with the highest concentration observed at 0 d (S). Thus, fermented milk of *S. thermophilus* CICC 6063 and *L. helveticus* CICC 6064 may have probiotic potential.

Aldehydes, with their lower threshold, have a significant impact on the flavor of fermented milk [36]. Among the ten aldehydes identified during fermentation and storage, only acetaldehyde was consistently present. Acetaldehyde is considered the most important flavor compound responsible for the fruity aroma in fermented milk, resembling green apple or nutty flavors [37]. The choice of starter cultures and pH level influence acetaldehyde production, with higher levels observed at 0 d (S) and 14 d (S) due to increased acidity [37,38]. Other aldehydes, like hexanal, are transient and easily converted into acidic compounds or alcohols in fermented milk [17]. The levels of hexanal detected in this study were minimal at both 4 h (F) and 21 d (S). Straight-chain aldehydes such as hexanal, heptanal, and nonanal, derived from the oxidation of unsaturated fatty acids in milk fat, contribute to the grassy and herbaceous aromas in fermented milk [39].

Ketones are formed through the thermal degradation of amino acids, the oxidation of unsaturated fatty acids, and the Maillard reaction. These compounds have a significant impact on the aroma of dairy products due to their low perception thresholds [40]. In this study, 11 ketones were identified, including acetoin, 2,3-butanedione, 2,3-pentanedione, 2-heptanone, and 2-nononone, which are important flavor components. Acetoin, known for its slightly creamy, slightly sweet, buttery flavor, when combined with diacetyl, gives sour milk a mild and pleasant buttery taste [41]. 2,3-Butanedione (diacetyl), responsible for the flavor of sour butter, is derived from the fermentation of citric acid [42]. It has been detected at levels ranging from 32.44 to 135.28 µg/L during fermentation and storage. The production of 2,3-butanedione and 2,3-pentanedione is a result of the chemical decarboxylation of their precursors, 2-acetolactate and 2-aceto-2-hydroxybutyrate. Acetoin, 2,3-butanedione, and 2,3-pentanedione, which are considered important odor compounds in yogurt, showed the highest levels in the samples, consistent with the findings of this study [43]. The stability of 2, 3-pentanedione and acetoin remains unchanged throughout fermentation and storage, as shown in Figure 5, indicating a potential enzymatic reduction of acetoin from 2, 3-pentanedione by diacetyl reductase [44]. However, it is important to note that this particular result has not been documented in the current literature.

Alcohols are the final byproducts of amino acid metabolism and glucose degradation in yogurts. The presence of alcohols in fermented milk has been associated with lactose metabolism, reduction of methyl ketones, and amino acid metabolism [45]. A comprehensive analysis found 11 different alcohols in the samples, with 1-heptanol being the only one detected during late storage. The presence of 1-heptanol significantly altered the flavor profile of the sample, as it is considered an important flavor component in fermented milk [46].

Esters can be formed in yogurt when alcohols react with free acids [15]. LAB contain various esters that can be used in the alcoholysis process to produce flavor esters directly from glycerol esters and alcohols [47]. For example, ethyl acetate and ethyl hexanoate can be synthesized by esterifying ethanol with acetic acid and hexanoate acid, respectively, using esterases. Esters, especially ethyl acetate, are important for developing fruity characteristics in dairy products [48]. They contribute to fruity and floral flavors in fermented milk while reducing the strong odors of fatty acids and amines [31]. The analyzed sample had ethyl caproate in concentrations ranging from 0.19 to 2.12 µg/L, with the highest levels detected at the end of fermentation. Ethyl caproate is a crucial flavor compound that gives the product a fruity taste.

The presence of dominant *Lactobacillus* species in fermented milk contributes to the production of various compounds, including esters, aldehydes, acids, ketones, and alcohols [15]. Among these species, *L. helveticus* is noteworthy for its strong lipolysis and proteolytic activity. This activity involves the breakdown of milk proteins into smaller peptides using peptides and proteinases. Through this enzymatic process, desirable flavor compounds are formed from milk peptides and fats [49]. Furthermore, a group of peptidases further degrade the resulting peptides into free amino acids following initial breakdown by rennet and bacterial proteases [50].

In addition to the strain ratio, the flavor of fermented milk can be influenced by various factors such as the processing conditions, milk source, and other ingredients used in the production process [51]. The presence of numerous volatile flavor compounds in the sample contributes to the distinct flavor of fermented milk. Fermented milk had the highest number of key flavor compounds detected upon completion of fermentation. Research on flavor during fermentation and storage can enhance the production of consistent yogurt products and improve consumer acceptance. Many studies have shown a strong correlation between lactic acid bacteria and flavor formation in fermented milk and other fermented foods [15]. Therefore, the combination of *S. thermophilus* CICC 6063 and *L. helveticus* CICC 6064 shows promising potential in fermented food production.

## 4. Materials and Methods

### 4.1. Materials

The strains *Streptococcus thermophilus* CICC 6063 and *Lactobacillus helveticus* CICC 6064 were sourced from the China Center of Industrial Culture Collection in Beijing, China. The UHT whole milk used in this study was obtained from Yili Industry Group Co., Ltd., located in Hohhot City, China. Additionally, the 2-Methyl-3-heptanone standard was purchased from Sigma-Aldrich (St. Louis, MO, USA).

### 4.2. Preparation of Fermented Milk

Freeze-dried cultures of *S. thermophilus* CICC 6063 and *L. helveticus* CICC 6064, stored at −80 °C, were utilized for this study. In order to create fermented milk, a standard initial inoculum size of 5 × 10 ^6^ CFU/mL was employed for all samples. The milk was pasteurized and subjected to heating at 90 °C for 30 min, followed by cooling to 42 °C. Various ratios of *S. thermophilus* CICC 6063 and *L. helveticus* CICC 6064 were added to the milk, including a control group with commercial cultures. The ratios of 1:1, 5:1, 10:1, 100:1, and 1000:1 (*S. thermophilus* CICC 6063 to *L. helveticus* CICC 6064) were selected and tested based on preliminary experiments and a literature review regarding the combination ratio of *Streptococcus* and *Lactobacillus*. The mixture was then incubated at 42 °C. Fermentation was terminated upon reaching a pH value of 4.6, and all fermented samples were stored at 4 °C for a duration of 21 days. Throughout the fermentation and refrigerated storage period, 50 mL samples were collected at specific time intervals for subsequent analysis.

### 4.3. pH Value, Titratable Acidity Determination, and Enumeration of Viable Cells

Throughout the fermentation process, the pH value of the samples was monitored using the *iCinac* fermentation monitor (Paris, France) lasting 24 h, in accordance with the ISO 26323:2009(E) method [52]. Additionally, the pH value was measured during the storage period using a pH meter from Mettler Toledo (Zurich, Switzerland). To evaluate the titratable acidity (TA), measurements were taken at various time points during the fermentation process. These time points included the initial point of fermentation (0 h (F)), two intermediate time points (2 h (F), 4 h (F)), the termination point of fermentation (0 d (S)), and different stages of refrigerated storage (1 d (S), 7 d (S), 14 d (S), and 21 d (S)). The protocols outlined in the GB5009.239-2016 guidelines [53] were followed for these measurements. To evaluate the count of viable cells, we consulted the guidelines outlined in GB4789.35-2016 [54].

### 4.4. Rheological Property Analysis

The gel formation of milk during fermentation was analyzed using a Rheolaser Master (Formulaction, Toulouse, France) equipped with multi-speckle diffusing wave spectroscopy (MS-DWS). In this study, 20 mL of inoculated milk samples were poured into glass tubes with inner diameters of 27.5 mm. These tubes were then placed in the pre-equilibrated Rheolaser Master apparatus at a temperature of 42 °C. The acidification process was allowed to proceed until the pH of the milk reached 4.6. Measurements were performed at 1 min intervals using the Rheolaser Master. The collected data were then analyzed using the accompanying software, TURBISCAN Lab, enabling the determination of various rheological parameters including the macroscopic viscosity index (MVI), solid–liquid balance (SLB), elastic index (EI), and fluidity index (FI).

### 4.5. Electronic Nose Analysis

For this investigation, we utilized the Fox 4000 E-nose system provided by ALPHA MOS in Toulouse, France. This system consists of three sensor matrix chambers and a total of 18 metal oxide sensors. To enhance sample differentiation, we selected 12 metal oxide sensors with high discriminative capability, namely, T30/1, P10/1, P10/2, P40/1, T70/2, PA/2, P30/1, P40/2, P30/2, T40/2, T40/1, and TA/2, based on preliminary experiments. The primary applications of these sensors are described in reference [55]. The response signal output from the sensors is denoted R (resistance value), and the maximum value was selected for data processing.

To conduct the measurements, each sample was accurately weighed to 5 g and sealed in 20 mL centrifugal bottles. These sealed bottles were incubated at a temperature of 60 °C for 5 min, and the headspace was injected into the electronic nose system. Throughout the measurement procedure, we maintained specific environmental conditions, including a sensor cleaning duration of 120 s, an internal flow rate of 150 mL/min, and a sample detection time of 120 s.

### 4.6. Sensory Evaluation

To evaluate the sensory characteristics of the different fermented milk samples, a group of 11 trained panelists used a modified 100-point intensity scale based on the guidelines outlined in the Chinese dairy industry guideline RHB 103-2004 [56]. The panelists rated the acidity, sweetness, viscosity, and denseness on a scale ranging from 0 to 15. The taste, flavor, and texture were evaluated on a separate 20-point intensity scale. To calculate the overall mean score for each sample, the highest and lowest scores were discarded, and the average of the remaining scores was calculated.

The sensory attributes of 10:1 and CSC were assessed using quantitative descriptive analysis (QDA). The flavor descriptors (creamy, fruity, cheesy, sour, and alcohol) were chosen in the preliminary tests by the panelists. The 5 descriptors were defined as follows, and relevant material objects or chemical standards were used as references [57]: cream with 35.5% fat content for “creamy”; 20 mg/kg ethyl hexanoate solution in distilled water for “fruity”; fresh butter for “cheesy”; 0.08% citric acid solution in distilled water for “sour”; 10 mg/kg n-butanol and 10 mg/kg ethyl caproate dissolved in distilled water for “alcohol”. In the study, 10 g of fermented milk samples were individually placed into 20 mL sensory evaluation cups and labeled with three random numbers. Each group member evaluated the samples for flavor attributes using a score scale ranging from 0 (absent) to 10 (very intense). Two training sessions were conducted prior to the formal experiment.

### 4.7. Analysis of Volatile Flavor Compounds by SPME-GC-MS

To extract the flavor compounds, a solid-phase microextraction (SPME) fiber (50/30 µm, DVB/CAR/PDMS, Agilent, Santa Clara, CA, USA) was used. The extraction process parameters were optimized. The procedure involved placing 10 g of the sample in a 40 mL headspace vial and adding 1 µL of 2-methyl-3-heptanone as an internal standard with a mass concentration of 0.816 mg/mL. After equilibrating at 45 °C in a water bath for 30 min, the SPME fiber was used for headspace extraction for 30 min. Then, it was desorbed for 5 min in GC-MS. A 7890B gas chromatograph equipped with a 5977A mass selective detector (MSD; Agilent, Santa Clara, CA, USA) was used to analyze the volatile flavour compounds of the samples.

GC: A DB-WAX column (60 m × 0.25 mm, 0.25 µm) was used. Helium gas was used as the carrier gas with a constant flow rate of 1.2 mL/min. The temperature program for the column started at 40 °C and was ramped up at a rate of 4 °C/min to 230 °C, which was then held for 2 min. The system was operated in split-less mode.

MS: An electron ionization (EI) source was applied at a voltage of 70 eV. The inlet temperature was set to 250 °C, while the ion source and quadrupole temperatures were maintained at 230 °C and 150 °C, respectively. The mass scan range used was 35 to 350 *m*/*z*, employing full scan mode.

### 4.8. Qualitative and Semi-Quantitative Analysis

The concentration of each compound was calculated using the following formula after the volatile flavor compounds were automatically identified using the National Institute of Standards Technology Mass Spectral Database 14 (accessed using MassHunter from Agilent Technologies, Inc.) Masshunter workstation. The retention indices (RIs) of volatile flavor compounds were calculated based on the retention time, which was determined by injection of a series of n-alkanes $C_{9}$–$C_{25}$ (from AccuStandard Inc., New Haven, CT, USA) under identical conditions. These RIs were then compared with the RI values reported in the literature (http://webbook.nist.gov/chemistry (accessed on 26 February 2024)). The relative abundances of all identified volatile compounds were compared using their peak areas in total ion chromatography (TIC) and then normalized by the peak areas of the internal standard (ISTD) from the same sample. Finally, the concentration of each compound was calculated using the following formula:

(1) $$c_{i}=\frac{A_{i}}{A_{s}}\times c_{s}$$

where “$c_{i}$” signifies the concentration (mg/L) of each compound, “$c_{s}$” denotes the concentration (mg/L) of the internal standard (ISTD), “$A_{i}$” indicates the chromatographic peak area for each compound, and “$A_{s}$” signifies the chromatographic peak area of the ISTD.

### 4.9. Statistical Analysis

The data were examined using Origin 2022 software from OriginLab Co., located in Northampton, MA, USA. All data were imported into the MetaboAnalyst 5.0 website (http://www.metaboanalyst.ca (accessed on 26 February 2024)) for statistical analysis. To determine the statistical significance between samples, a one-tailed Student’s *t*-test (*p* < 0.05) was performed. The variable importance on projection (VIP) score was utilized to identify important metabolite features in the PLS-DA. Volatile compounds that had both *p* < 0.05 and VIP > 1 were considered significantly different volatile compounds for variable selection [58].

## 5. Conclusions

Exploring different combinations of starter strains is essential in the fermented milk manufacturing industry to achieve desired qualities such as strong fermentation capability, distinct flavors, and probiotic properties. In this study, we focused on the influence of different inoculation ratios of *Streptococcus thermophilus* CICC 6063 and *Lactobacillus helveticus* CICC 6064 strains on fermented milk properties. The 10:1 ratio showed a viable count of 9.64 log10 CFU/mL, a fermentation time of 4.4 h, and a sensory evaluation score of 79.1 points. Throughout fermentation and storage, a total of 56 volatile compounds including carboxylic acids, alcohols, esters, ketones, aldehydes, and aromatic carbohydrates were identified. Seven discriminatory volatile metabolites were identified for distinguishing between different time points, including 2,3-butanedione, acetoin, 2,3-pentanedione, hexanoic acid, acetic acid, acetaldehyde, and butanoic acid. Significant flavor differences were observed between the samples stored for 21 d and the samples stored at other time points. A synergistic relationship was observed between 2,3-pentanedione and acetoin, as their concentrations exhibited similar patterns of change during fermentation and storage. This study provides insights into the synergistic effects of *Streptococcus thermophilus* and *Lactobacillus helveticus* strains in fermented milk production. Moreover, these findings have practical implications in improving the flavor and odor of dairy products during the fermentation and storage processes.

## Figures and Tables

**Table 1:** Volatile compounds produced in milk fermented by *S. thermophilus* CICC 6063 and *L. helveticus* CICC 6064 during fermentation and storage.

Volatile Compound	RT ^1^	RI ^2^	RI ^3^	Method ^4^	µg/L							
					0 h (F) ^5^	2 h (F)	4 h (F)	0 d (S) ^6^	1 d (S)	7 d (S)	14 d (S)	21 d (S)
Carboxylic acid compounds												
Acetic acid	23.51	1408	1461	MS, RI	0.39 ± 0.03	4.25 ± 2.92	27.68 ± 7.06	39.44 ± 12.65	12.71 ± 1.22	34.53 ± 2.51	42.38 ± 0.91	35.62 ± 0.93
Propanoic acid	27.111	1516	1540	MS, RI	-	-	0.18 ± 0	0.32 ± 0.1	0.11 ± 0	0.17 ± 0.05	0.17 ± 0	0.16 ± 0.01
Butanoic acid	30.655	1605	1647	MS, RI	-	4.19 ± 4.17	34.75 ± 6.65	50.45 ± 14.54	15.42 ± 1.17	25.69 ± 4.15	26.67 ± 1.1	31.56 ± 0.82
2-Methyl-hexanoic acid	32.183	1645	nf	MS	-	-	-	-	0.09 ± 0	-	-	0.34 ± 0
2-Methyl-butanoic acid	32.242	1647	1652	MS, RI	-	-	-	-	-	0.15 ± 0	-	0.4 ± 0.15
Hexanoic acid	38.709	1822	1861	MS, RI	-	13.85 ± 0	60.05 ± 11.77	90.84 ± 35.89	28.92 ± 3.44	37 ± 3.63	37.12 ± 3.47	55.67 ± 3.65
Pentanoic acid	38.735	1822	1762	MS, RI	-	2.84 ± 0.62	-	0.54 ± 0.22	0.28 ± 0.2	-	0.12 ± 0.05	0.21 ± 0.01
Octanoic acid	45.911	2036	2072	MS, RI	-	3.06 ± 2.6	10.81 ± 3.11	14.34 ± 6.93	6.8 ± 0.85	4.97 ± 0.83	5.71 ± 0.68	6.34 ± 1.45
n-Decanoic acid	52.46	2248	2314	MS, RI	-	-	1.15 ± 0.41	2.47 ± 0.35	-	-	-	-
Benzoic acid	57.177	2413	2457	MS, RI	-	-	3.63 ± 0	3.3 ± 0.48	-	7.41 ± 0	-	-
Aldehydes												
Acetaldehyde	3.497	STD	714	MS	-	5.59 ± 0	20.26 ± 5.27	74.39 ± 56.32	8.35 ± 5.89	5.93 ± 0.58	41.32 ± 10.56	35.62 ± 0
Pentanal	8.955	1056	1100	MS, RI	-	-	0.31 ± 0	1.11 ± 0.33	-	-	-	-
Hexanal	9.087	1061	1079	MS, RI	-	-	0.78 ± 0.27	-	-	-	-	2.4 ± 0.23
3-Methyl-pentanal	9.207	1065	nf	MS	1.1 ± 0	-	-	-	-	-	-	1.23 ± 0
2-Methyl-2-butenal	13.622	1195	1104	MS, RI	0.15 ± 0	-	-	-	-	0.31 ± 0	0.72 ± 0	1.11 ± 0
2-Methyl-pentanal	21.114	1370	nf	MS	6.26 ± 0	-	2.32 ± 0	-	-	-	-	-
2-Methyl-hexanal	21.306	1375	nf	MS	-	-	-	-	-	-	4.77 ± 0	-
Nonanal	23.814	1410	1390	MS, RI	-	-	-	-	-	-	-	0.76 ± 0
Furfural	23.865	1410	1468	MS, RI	-	-	0.22 ± 0	-	-	-	-	-
Benzaldehyde	26.316	1428	1520	MS, RI	-	-	-	1.94 ± 0	-	-	-	-
Ketones												
Acetone	4.237	STD	814	MS	-	18.81 ± 0	23.45 ± 0	42.99 ± 0	2.51 ± 0	14.85 ± 0	13.63 ± 0	22.63 ± 0
2-Butanone	4.994	STD	881	MS	-	2.06 ± 1.36	2.04 ± 0.86	4.02 ± 2.56	-	1.42 ± 0	-	-
2,3-Butanedione	6.418	STD	971	MS	-	52.39 ± 0	49.75 ± 36.67	111.51 ± 9.48	32.44 ± 0	129 ± 1.94	122.87 ± 6.89	135.28 ± 24.24
2,3-Pentanedione	8.311	1033	1062	MS, RI	-	6.08 ± 1.85	12.93 ± 2.91	21.8 ± 2.99	9.18 ± 0.35	37.25 ± 5.72	45.01 ± 0.47	51.36 ± 3.78
2-Heptanone	12.604	1167	1184	MS, RI	64.9 ± 26.76	108.5 ± 0	28.79 ± 2.37	41.34 ± 13.74	22.85 ± 0.23	31.77 ± 2.88	28.91 ± 2.84	39.17 ± 5.93
Acetoin	16.66	1267	1280	MS, RI	-	76.26 ± 28.11	153.22 ± 38.03	231.14 ± 45.89	58.65 ± 3.37	109.48 ± 19.04	97.74 ± 1.95	104.88 ± 6.01
Cyclohexanone	16.945	1274	1282	MS, RI	-	-	-	-	-	-	-	0.73 ± 0
2-Hydroxy-3-pentanone	19.702	1338	1380	MS, RI	-	3.21 ± 0	4.95 ± 0.79	6.57 ± 1.61	2.42 ± 0.25	4.19 ± 0.03	4.18 ± 0	5.69 ± 0.26
4-Methyl-2-hexanone	21.205	1372	nf	MS	-	-	-	-	-	1.39 ± 0	3.03 ± 0	1.02 ± 0
2-Nonanone	21.441	1378	1386	MS, RI	2.91 ± 0	1.46 ± 0	-	2.99 ± 0	4.57 ± 0.92	3.99 ± 1.55	3.95 ± 0	7.79 ± 0.92
Alcohols												
Methyl alcohol	3.669	STD	888	MS	-	97.33 ± 0	-	20.41 ± 0	-	-	8.35 ± 2.54	-
2-Methyl-1-pentanol	17.213	1281	nf	MS	-	-	-	-	-	-	-	0.19 ± 0
3-Penten-2-ol	18.304	1306	1170	MS, RI	-	-	0.82 ± 0	-	-	-	-	-
3-Pentanol	19.017	1323	1108	MS, RI	-	-	4.17 ± 1.34	6.29 ± 1.7	2.39 ± 0.18	3.86 ± 0.78	4.09 ± 0.1	5.48 ± 0.4
2-Hexanol	19.721	1339	1226	MS, RI	-	-	-	-	-	3.47 ± 0	-	-
1,2-Ethanediol	20.355	1353	1621	MS, RI	-	-	-	1.61 ± 0.32	0.43 ± 0.05	0.58 ± 0	0.64 ± 0.08	0.83 ± 0.03
Benzaldehyde	26.316	1428	1520	MS, RI	-	-	-	1.94	-	-	-	-
Linalool	27.563	1527	1549	MS, RI	-	-	-	-	-	-	-	0.18 ± 0
1-Propene-1-thiol	41.706	1908	nf	MS	-	-	-	-	0.05 ± 0	0.03 ± 0	2.58 ± 0	0.06 ± 0
1,4-Butanediol	41.75	1909	nf	MS	-	-	-	0.77 ± 0	-	-	-	-
3-Methyl-3-hexanol	43.237	1954	nf	MS	-	-	-	-	-	0.02 ± 0	0.02 ± 0	-
Esters												
Ethyl acetate	8.77	1049	nf	MS	-	0.16 ± 0	-	-	-	-	-	-
Ethyl hexanoate	14.494	1216	1246	MS, RI	-	-	-	2.12 ± 0	-	0.19 ± 0	-	0.36 ± 0
Ethyl orthoformate	29.256	1570	nf	MS	-	-	-	-	-	0.04 ± 0	-	-
δ-Nonalactone	50.4	2179	nf	MS	-	-	-	-	0.42 ± 0.1	-	-	0.35 ± 0.01
2H-Pyran-2-one, tetrahydro-6-pentyl-	50.435	2180	1999	MS, RI	-	-	-	-	-	-	0.21 ± 0.01	0.37 ± 0
Others												
Toluene	7.28	STD	1037	MS	-	2.09 ± 0	-	-	-	-	-	1.32 ± 0
*p*-Xylene	10.532	1110	1164	MS, RI	-	29.83 ± 30.25	12.41 ± 5.51	18.13 ± 3.89	8.74 ± 1.93	14.87 ± 3.51	13.86 ± 3.21	17.04 ± 4.02
1,3-Dimethyl-benzene	10.919	1121	1141	MS, RI	-	12.01 ± 0	8.28 ± 4.67	16.18 ± 9.71	-	-	8.67 ± 0	11.84 ± 4.84
Limonene	13.047	1179	1198	MS, RI	-	2.91 ± 0	-	8.86 ± 0	-	-	-	-
1-Ethyl-4-methyl-benzene	14.106	1207	1216	MS, RI	-	-	-	-	-	-	-	1.39 ± 0.5
Styrene	15.405	1238	1259	MS, RI	1.8 ± 1.29	20.24 ± 11.45	14.34 ± 4.11	21.29 ± 7.97	14.99 ± 0.97	18.22 ± 4.9	16.79 ± 2.2	22.01 ± 3.41
Mesitylene	16.363	1261	1237	MS, RI	-	-	-	-	-	-	-	1.14
Dimethyl sulfone	40.667	1878	1912	MS, RI	-	0.42 ± 0.26	0.35 ± 0.28	0.91 ± 0.36	0.22 ± 0.15	0.59 ± 0.11	0.41 ± 0.03	0.21 ± 0.02
Butylated Hydroxytoluene	40.851	1883	1920	MS, RI	0.31 ± 0	-	-	-	-	0.32 ± 0	0.1 ± 0	-
Phenol	43.943	1975	1987	MS, RI	0.06 ± 0	-	-	-	0.1 ± 0.04	-	-	0.1 ± 0
Internal standard												
3-Heptanone, 2-methyl-	11.987	1150	nf	MS	81.60	81.60	81.60	81.60	81.60	81.60	81.60	81.60

^1^ RT, retention time. ^2^ RI, retention index. The RI of unknown compounds in a DB-Wax column calculated against the GC-MS retention time of n-alkanes (*C*_9_–*C*_25_). ^3^ RI from a database (http://webbook.nist.gov/chemistry, accessed on 26 February 2024). ^4^ RI, agreed with the retention index from the literature; MS, compared with NIST 14 Mass Spectral Database; STD, agreed with the mass spectrum of standard chemical; nf, not found; “-”, not detected. ^5^ F, fermentation. ^6^ S, storage. Data are expressed as mean ± SD.

## Data Availability

Data are contained within the article.
